# Supplementary material for: Influence of Solvent Polarity on the Conformer Ratio of Bicalutamide in Saturated Solutions: Insights from NOESY NMR Analysis and Quantum-Chemical Calculations
Source: Int J Mol Sci. 2024 Jul 28;25(15):8254. doi: 10.3390/ijms25158254 (PMC11311660; doi:10.3390/ijms25158254)
Supplement: Supplementary file 1 [file ijms-25-08254-s001.zip › ijms-3093546-supplementary.pdf]

Supplementary Materials

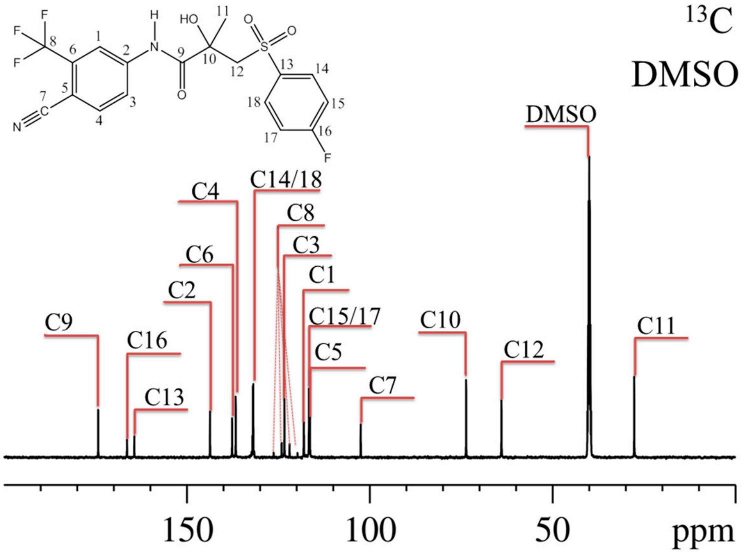

Figure S1. ^{13}C NMR spectrum of BCL in DMSO-d_6

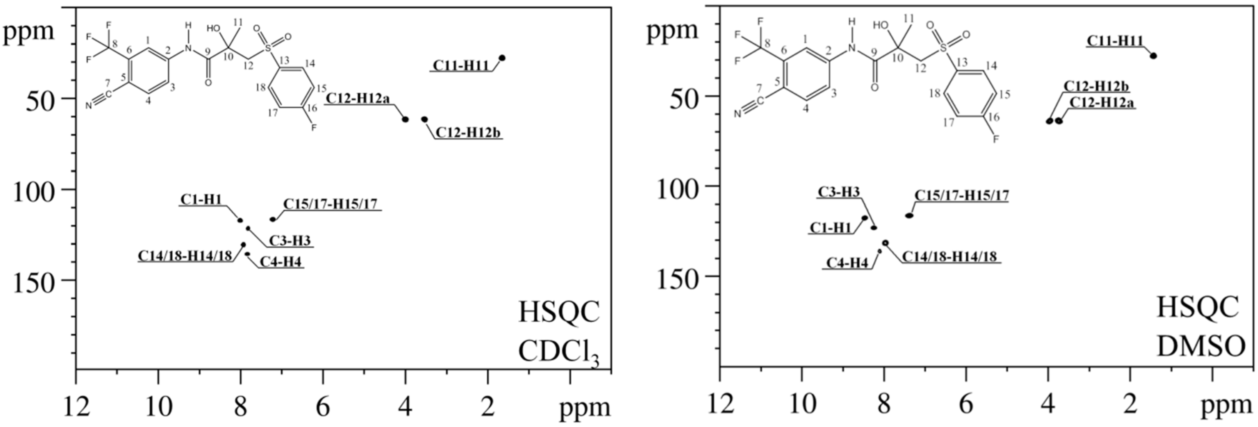

a

b

Figure S2. ^{1}H-^{13}C HSQC spectra of BCL recorded in CDCl$_3$ (a) and DMSO-d_6 (b)

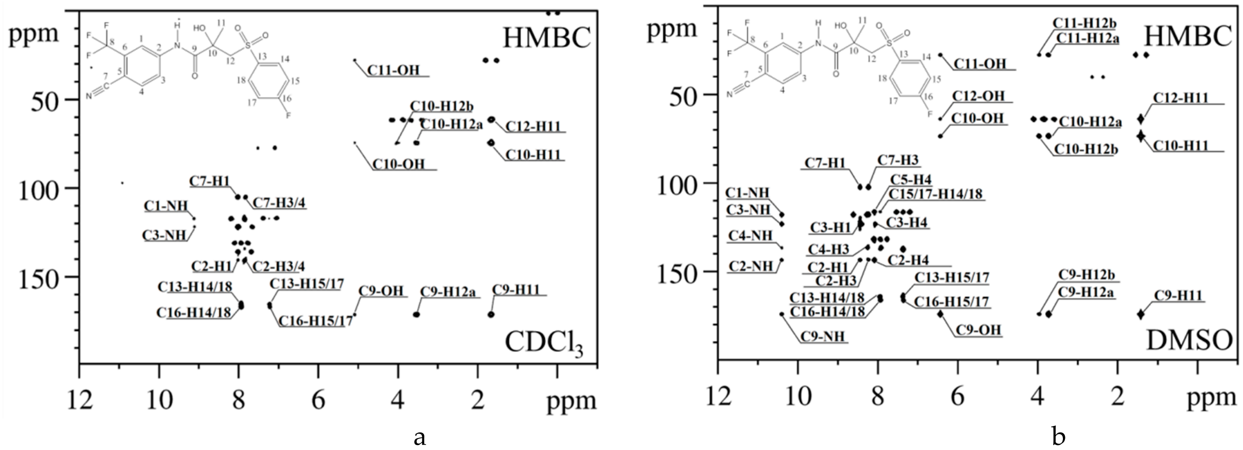

a

b

Figure S3. ^{1}H-^{13}C HMBC spectra of BCL recorded in CDCl$_3$ (a) and DMSO-d_6 (b)

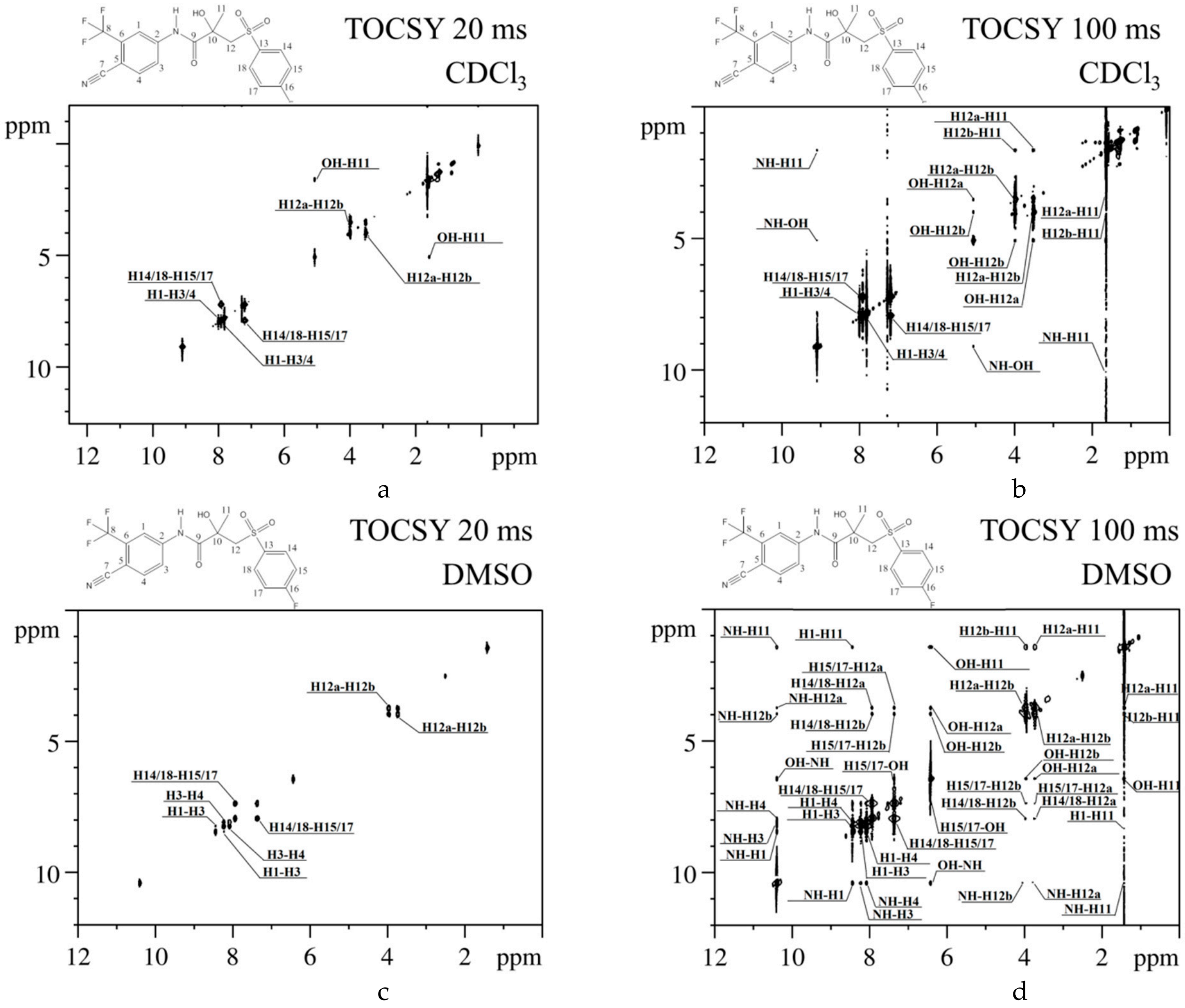

Figure S4. ^{1}H-^{1}H TOCSY spectra of BCL recorded in $CDCl_3$ (a, b) and DMSO-d_6 (c, d)

Cartesian coordinates of BCL-1

6	-1.446679000	-0.808833000	-0.542724000
6	-0.664508000	-1.543754000	0.352074000
6	-1.234968000	-2.015840000	1.534864000
6	-2.557948000	-1.752233000	1.824955000
6	-3.344041000	-1.007861000	0.943953000
6	-2.766049000	-0.538572000	-0.241299000
6	-4.708665000	-0.770299000	1.288609000
6	-3.595985000	0.258024000	-1.221012000
6	1.484478000	-1.311973000	-0.850984000
6	2.977633000	-1.652858000	-0.630755000
6	3.360633000	-2.679312000	-1.690665000
6	3.792844000	-0.375147000	-0.861148000
6	2.020363000	1.446103000	0.443604000
6	1.201318000	1.105303000	1.510341000
6	-0.115814000	1.533218000	1.518729000
6	-0.572721000	2.279064000	0.445722000
6	0.237807000	2.632942000	-0.621788000
6	1.555723000	2.214759000	-0.616075000
9	-4.541513000	-0.512070000	-1.784645000
9	-2.848133000	0.762734000	-2.212915000

9	-4.216924000	1.282387000	-0.625698000
1	2.217584000	2.482161000	-1.429363000
1	-0.172050000	3.217570000	-1.434060000
1	-0.791022000	1.288186000	2.327406000
1	1.600776000	0.518081000	2.326237000
1	3.496339000	0.101409000	-1.793173000
1	4.861350000	-0.597676000	-0.890082000
1	2.800809000	-3.600502000	-1.525187000
1	3.133856000	-2.305070000	-2.687904000
1	4.423986000	-2.905713000	-1.613956000
1	3.510000000	-1.535320000	1.245418000
1	-0.635901000	-2.588156000	2.233861000
1	-1.006725000	-0.440211000	-1.454473000
1	-2.999249000	-2.120040000	2.741780000
1	1.204500000	-2.267311000	0.864784000
9	-1.851154000	2.664796000	0.434642000
16	3.694332000	0.877125000	0.427388000
8	3.924572000	0.177138000	1.691590000
8	4.560953000	1.959131000	0.026718000
8	3.204250000	-2.232011000	0.638176000
8	1.118539000	-0.694725000	-1.831595000
7	0.683972000	-1.791312000	0.138782000
7	-5.804128000	-0.610785000	1.608439000

Cartesian coordinates of BCL-2

6	1.584755000	-1.341548000	-0.112967000
6	0.639646000	-1.045999000	0.872195000
6	1.000220000	-0.239725000	1.951880000
6	2.275924000	0.289920000	2.008240000
6	3.214576000	0.029202000	1.011173000
6	2.853353000	-0.807852000	-0.051806000
6	4.504667000	0.631413000	1.108211000
6	3.837338000	-1.094763000	-1.158146000
6	-1.788088000	-1.114959000	1.289329000
6	-3.064791000	-1.649247000	0.599493000
6	-3.758677000	-2.580282000	1.586511000
6	-3.974341000	-0.445014000	0.328064000
6	-1.938786000	1.358716000	-0.605726000
6	-0.828673000	1.077721000	-1.389416000
6	0.385035000	1.676862000	-1.092073000
6	0.448735000	2.526631000	-0.001884000
6	-0.653852000	2.817234000	0.787116000
6	-1.866109000	2.231120000	0.473014000
9	4.180954000	0.034239000	-1.800056000
9	3.326208000	-1.931403000	-2.075818000
9	4.961060000	-1.651045000	-0.691197000
1	-2.746120000	2.451558000	1.062631000
1	-0.549023000	3.491434000	1.626288000
1	1.273476000	1.489854000	-1.679841000
1	-0.924462000	0.404954000	-2.231296000
1	-4.057812000	0.174914000	1.218181000
1	-4.969988000	-0.770454000	0.019471000
1	-3.125842000	-3.448349000	1.774213000

1	-3.944553000	-2.070096000	2.530474000
1	-4.700567000	-2.927788000	1.162370000
1	-2.904747000	-1.821041000	-1.342837000
1	0.274794000	-0.014210000	2.716995000
1	1.314565000	-1.971500000	-0.950427000
1	2.556162000	0.930826000	2.833609000
1	-0.825676000	-2.112040000	-0.126701000
9	1.621830000	3.087901000	0.308281000
16	-3.496222000	0.631224000	-1.032806000
8	-3.257561000	-0.247680000	-2.178118000
8	-4.495722000	1.669033000	-1.111338000
8	-2.762551000	-2.389234000	-0.565489000
8	-1.857535000	-0.391184000	2.263434000
7	-0.645174000	-1.538783000	0.687921000
7	5.533780000	1.137507000	1.219750000

Cartesian coordinates of BCL-3

6	-3.135558000	0.472453000	-0.381118000
6	-2.155103000	0.481471000	0.616205000
6	-2.340617000	-0.282976000	1.768988000
6	-3.491765000	-1.036697000	1.906287000
6	-4.474977000	-1.050108000	0.917637000
6	-4.279935000	-0.280377000	-0.236744000
6	-5.640930000	-1.847402000	1.121486000
6	-5.325009000	-0.273586000	-1.324640000
6	0.066952000	1.411442000	1.177490000
6	1.148222000	2.318279000	0.534154000
6	1.266538000	3.562566000	1.405036000
6	2.475226000	1.552237000	0.572591000
6	4.058920000	-0.553951000	-0.411682000
6	4.244602000	-1.551900000	0.536434000
6	5.501326000	-2.112879000	0.687947000
6	6.531059000	-1.655493000	-0.119694000
6	6.353932000	-0.666762000	-1.074890000
6	5.095179000	-0.108022000	-1.221044000
9	-5.526863000	-1.503963000	-1.817544000
9	-4.972871000	0.514833000	-2.353101000
9	-6.503836000	0.173571000	-0.866857000
1	4.907577000	0.652268000	-1.968092000
1	7.189707000	-0.357946000	-1.688231000
1	5.692504000	-2.896719000	1.408434000
1	3.407402000	-1.892731000	1.131995000
1	2.637330000	1.144719000	1.568721000
1	3.307080000	2.189785000	0.274149000
1	2.069649000	4.198567000	1.032621000
1	0.333738000	4.125561000	1.357656000
1	1.458924000	3.289032000	2.441058000
1	1.234740000	2.175059000	-1.424723000
1	-1.585317000	-0.280732000	2.538806000
1	-2.997283000	1.060143000	-1.279174000
1	-3.639851000	-1.632264000	2.797489000
1	-0.955271000	1.748688000	-0.490928000
9	7.745463000	-2.196114000	0.023823000

16	2.457406000	0.169643000	-0.575359000
8	1.462190000	-0.783217000	-0.139586000
8	2.357970000	0.761793000	-1.908104000
8	0.790240000	2.736985000	-0.769668000
8	0.231185000	0.944881000	2.286895000
7	-1.030467000	1.262506000	0.392531000
7	-6.566319000	-2.503354000	1.325490000

Cartesian coordinates of BCL-4

6	-2.754431000	0.065362000	0.434141000
6	-2.250931000	1.156682000	-0.279829000
6	-2.971276000	1.658728000	-1.365300000
6	-4.170413000	1.085410000	-1.733376000
6	-4.682423000	-0.004052000	-1.027340000
6	-3.954984000	-0.503680000	0.059515000
6	-5.928030000	-0.560717000	-1.446428000
6	-4.489603000	-1.683150000	0.836036000
6	-0.157257000	1.438587000	1.007443000
6	1.119566000	2.317946000	0.979553000
6	1.137408000	3.125601000	2.271105000
6	2.326148000	1.373091000	0.962522000
6	3.888207000	-0.493735000	-0.448897000
6	3.724920000	-1.782524000	0.043016000
6	4.838456000	-2.589999000	0.200880000
6	6.080118000	-2.078261000	-0.143005000
6	6.252352000	-0.796989000	-0.643093000
6	5.135357000	0.007087000	-0.797367000
9	-4.641130000	-2.757792000	0.046111000
9	-5.690011000	-1.409103000	1.371476000
9	-3.676740000	-2.037055000	1.839770000
1	5.221440000	1.006845000	-1.202801000
1	7.243202000	-0.453901000	-0.908747000
1	4.759969000	-3.602079000	0.574497000
1	2.734113000	-2.148311000	0.280401000
1	2.206011000	0.609321000	1.728618000
1	3.258713000	1.919291000	1.101962000
1	2.057806000	3.706584000	2.330368000
1	0.294404000	3.817448000	2.275407000
1	1.056082000	2.468764000	3.135490000
1	1.665262000	2.883373000	-0.823605000
1	-2.585516000	2.502420000	-1.925777000
1	-2.198767000	-0.325454000	1.270594000
1	-4.726094000	1.475547000	-2.575642000
1	-0.724166000	2.519699000	-0.558288000
9	7.157092000	-2.856154000	0.007402000
16	2.462268000	0.531713000	-0.619506000
8	1.298288000	-0.305199000	-0.802053000
8	2.762501000	1.575672000	-1.597915000
8	1.116901000	3.231507000	-0.102334000
8	-0.298759000	0.582906000	1.857405000
7	-1.048622000	1.770812000	0.038507000
7	-6.935976000	-0.976157000	-1.820150000

Cartesian coordinates of BCL-5

6	3.454520000	-0.000630000	-0.548034000
6	2.533385000	-0.427787000	0.414166000
6	2.905281000	-0.448270000	1.759355000
6	4.178421000	-0.044014000	2.118594000
6	5.101527000	0.385087000	1.165989000
6	4.720256000	0.401153000	-0.182139000
6	6.398334000	0.791544000	1.602046000
6	5.696499000	0.860189000	-1.237353000
6	0.209046000	-1.244680000	0.682372000
6	-1.002531000	-1.623120000	-0.194013000
6	-1.211508000	-3.130569000	-0.051922000
6	-2.171368000	-0.793235000	0.358942000
6	-4.733174000	0.232927000	-0.152567000
6	-5.567814000	0.231842000	0.956713000
6	-6.349475000	1.345642000	1.216323000
6	-6.269030000	2.425486000	0.351757000
6	-5.445701000	2.433695000	-0.763740000
6	-4.666631000	1.317522000	-1.017338000
9	5.163234000	0.808688000	-2.468626000
9	6.798488000	0.097226000	-1.248327000
9	6.085259000	2.125628000	-1.024730000
1	-4.026318000	1.274913000	-1.888890000
1	-5.432448000	3.298182000	-1.413828000
1	-7.018678000	1.386833000	2.065172000
1	-5.614195000	-0.641006000	1.594861000
1	-1.990751000	0.267083000	0.180422000
1	-2.314926000	-0.972932000	1.422263000
1	-2.063765000	-3.458390000	-0.645712000
1	-0.320672000	-3.648842000	-0.406761000
1	-1.387304000	-3.395045000	0.989349000
1	-1.547168000	-1.328207000	-2.031360000
1	2.199495000	-0.777324000	2.505518000
1	3.174687000	0.016217000	-1.593306000
1	4.471377000	-0.058391000	3.160147000
1	1.100482000	-0.767147000	-1.035178000
9	-7.022511000	3.502319000	0.598904000
16	-3.722748000	-1.185894000	-0.461299000
8	-3.434829000	-1.228566000	-1.892924000
8	-4.327073000	-2.328576000	0.185654000
8	-0.718718000	-1.259951000	-1.533185000
8	0.161851000	-1.355082000	1.891318000
7	1.278820000	-0.815406000	-0.040719000
7	7.433159000	1.111737000	1.995188000

Cartesian coordinates of BCL-6

6	1.172464000	-1.381205000	0.318300000
6	0.276566000	-0.757725000	1.191422000
6	0.729591000	0.249830000	2.042062000
6	2.054298000	0.642048000	1.984128000
6	2.948244000	0.047071000	1.095735000
6	2.488942000	-0.982398000	0.263878000
6	4.293074000	0.523028000	1.055696000

6	3.418189000	-1.610011000	-0.743758000
6	-2.142033000	-0.486349000	1.577282000
6	-3.459936000	-1.020417000	0.971922000
6	-4.574629000	-0.896910000	2.001493000
6	-3.811919000	-0.159202000	-0.242931000
6	-1.352752000	1.135727000	-1.056094000
6	-0.074758000	0.903068000	-1.551018000
6	0.959203000	1.762846000	-1.222179000
6	0.687174000	2.826360000	-0.379619000
6	-0.580734000	3.076108000	0.117286000
6	-1.614420000	2.225290000	-0.237794000
9	3.831503000	-0.697750000	-1.642590000
9	2.822699000	-2.597323000	-1.428462000
9	4.509304000	-2.124335000	-0.164230000
1	-2.606502000	2.414734000	0.147044000
1	-0.743231000	3.918511000	0.775880000
1	1.965429000	1.605306000	-1.586690000
1	0.106182000	0.045210000	-2.184892000
1	-4.082526000	0.850973000	0.056835000
1	-4.664752000	-0.600547000	-0.761769000
1	-4.374282000	-1.550241000	2.854369000
1	-4.636591000	0.121346000	2.379102000
1	-5.527046000	-1.182826000	1.554942000
1	-3.456598000	-2.947528000	1.244756000
1	0.041989000	0.732547000	2.717525000
1	0.819865000	-2.154710000	-0.351305000
1	2.408884000	1.435581000	2.628480000
1	-1.273984000	-1.874596000	0.437178000
9	1.689467000	3.637262000	-0.019034000
16	-2.623172000	0.011178000	-1.599311000
8	-1.973049000	-1.264701000	-1.829961000
8	-3.376956000	0.643999000	-2.659044000
8	-3.322421000	-2.351884000	0.503618000
8	-2.127314000	0.475661000	2.318725000
7	-1.050842000	-1.162735000	1.122284000
7	5.367373000	0.939757000	1.046973000

Cartesian coordinates of BCL-7

6	-1.149387000	-1.136536000	-0.327883000
6	-0.214101000	-1.359285000	0.684574000
6	-0.614170000	-1.298004000	2.020398000
6	-1.923571000	-1.009456000	2.341821000
6	-2.864306000	-0.771055000	1.338406000
6	-2.455928000	-0.837784000	0.001525000
6	-4.205808000	-0.473506000	1.724667000
6	-3.456906000	-0.591017000	-1.102228000
6	1.794285000	-1.464991000	-0.748512000
6	3.327603000	-1.469383000	-0.556549000
6	3.984120000	-2.148315000	-1.750193000
6	3.801983000	-0.016754000	-0.488570000
6	1.454594000	1.554057000	0.130383000
6	0.464256000	1.738916000	1.087471000
6	-0.789271000	2.184043000	0.703069000

6	-1.024683000	2.414002000	-0.641729000
6	-0.052926000	2.219729000	-1.609178000
6	1.205221000	1.798736000	-1.212791000
9	-4.388276000	-1.558461000	-1.133547000
9	-2.873371000	-0.558366000	-2.308386000
9	-4.094873000	0.573650000	-0.934218000
1	1.972476000	1.657626000	-1.961130000
1	-0.288918000	2.399206000	-2.649237000
1	-1.582209000	2.341110000	1.421715000
1	0.680765000	1.533267000	2.127040000
1	3.699947000	0.473833000	-1.454121000
1	4.854689000	0.007130000	-0.201775000
1	3.709059000	-3.205612000	-1.781499000
1	3.645804000	-1.696569000	-2.680138000
1	5.068715000	-2.068258000	-1.675197000
1	3.767962000	-3.027691000	0.523799000
1	0.112655000	-1.457533000	2.808232000
1	-0.840634000	-1.176117000	-1.359071000
1	-2.233162000	-0.958624000	3.377293000
1	1.741935000	-1.664493000	1.233007000
9	-2.234129000	2.834744000	-1.022439000
16	3.069733000	1.104740000	0.730430000
8	2.870371000	0.393413000	1.978735000
8	3.914422000	2.278252000	0.690300000
8	3.701990000	-2.080696000	0.668162000
8	1.291786000	-1.283549000	-1.839193000
7	1.127767000	-1.607371000	0.429854000
7	-5.273990000	-0.237911000	2.087203000

Cartesian coordinates of BCL-8

6	-2.252259000	-0.062509000	0.367086000
6	-1.901519000	1.136254000	-0.264413000
6	-2.820065000	1.768359000	-1.102521000
6	-4.064353000	1.196020000	-1.299761000
6	-4.420529000	-0.000671000	-0.679975000
6	-3.491887000	-0.626532000	0.162625000
6	-5.717654000	-0.541694000	-0.929201000
6	-3.846258000	-1.925040000	0.844786000
6	0.005864000	2.677667000	-0.562568000
6	1.449357000	2.937855000	-0.075097000
6	1.423437000	3.422989000	1.374555000
6	2.360954000	1.709780000	-0.312413000
6	3.350134000	-0.844876000	0.217076000
6	2.612854000	-1.813642000	-0.451625000
6	3.276007000	-2.850012000	-1.086012000
6	4.661050000	-2.881617000	-1.028709000
6	5.405760000	-1.925001000	-0.357368000
6	4.736432000	-0.890651000	0.275527000
9	-2.841188000	-2.379327000	1.609124000
9	-4.129294000	-2.883378000	-0.050867000
9	-4.918884000	-1.785673000	1.636111000
1	5.278107000	-0.133759000	0.827827000
1	6.484182000	-2.005043000	-0.332539000

1	2.742798000	-3.630841000	-1.611436000
1	1.531727000	-1.766317000	-0.455261000
1	3.378777000	2.073024000	-0.451476000
1	2.063772000	1.161850000	-1.206991000
1	2.434425000	3.670902000	1.693485000
1	0.808009000	4.320763000	1.429614000
1	1.022384000	2.676146000	2.059549000
1	1.225076000	4.236412000	-1.451704000
1	-2.556300000	2.692482000	-1.591698000
1	-1.542485000	-0.552289000	1.021054000
1	-4.781276000	1.681779000	-1.948527000
1	-0.062260000	1.050315000	0.633632000
9	5.303891000	-3.881094000	-1.640201000
16	2.510196000	0.492904000	1.010268000
8	3.361104000	1.032107000	2.045135000
8	1.168845000	0.021044000	1.358523000
8	1.974702000	3.936640000	-0.909680000
8	-0.473534000	3.429208000	-1.396244000
7	-0.623779000	1.621561000	0.000201000
7	-6.772461000	-0.945662000	-1.157507000

Cartesian coordinates of BCL-9

6	-3.232618000	0.025648000	-0.264025000
6	-2.576813000	-0.726934000	0.714747000
6	-3.249465000	-1.055510000	1.893660000
6	-4.549515000	-0.641617000	2.095322000
6	-5.212975000	0.111116000	1.125325000
6	-4.534366000	0.436475000	-0.054830000
6	-6.557229000	0.516833000	1.381938000
6	-5.234053000	1.250405000	-1.117781000
6	-0.410567000	-0.981765000	-0.465069000
6	0.949796000	-1.680273000	-0.264281000
6	1.048327000	-2.788558000	-1.312341000
6	1.999313000	-0.573937000	-0.450182000
6	4.655596000	0.206931000	0.007679000
6	5.208394000	0.935915000	-1.036462000
6	5.961461000	2.062847000	-0.750446000
6	6.136905000	2.421079000	0.576551000
6	5.596834000	1.695388000	1.627233000
6	4.844590000	0.570408000	1.334581000
9	-5.612224000	2.446959000	-0.641486000
9	-6.338204000	0.627602000	-1.557628000
9	-4.449780000	1.472524000	-2.179761000
1	4.422936000	-0.035805000	2.125848000
1	5.777155000	2.011932000	2.645766000
1	6.415855000	2.657752000	-1.531268000
1	5.062524000	0.609303000	-2.057885000
1	1.914200000	0.157837000	0.353582000
1	1.887645000	-0.082466000	-1.414328000
1	1.997327000	-3.316221000	-1.226519000
1	0.238663000	-3.501115000	-1.154752000
1	0.967659000	-2.373388000	-2.315469000
1	1.914629000	-2.463108000	1.224449000

1	-2.748589000	-1.640593000	2.656356000
1	-2.717615000	0.280774000	-1.175633000
1	-5.068178000	-0.897725000	3.009541000
1	-0.867647000	-1.704971000	1.335291000
9	6.863820000	3.508043000	0.856671000
16	3.676559000	-1.216942000	-0.370805000
8	3.722023000	-2.112310000	0.782930000
8	4.062143000	-1.704862000	-1.675620000
8	0.997363000	-2.201877000	1.052035000
8	-0.643386000	-0.351857000	-1.476938000
7	-1.269017000	-1.174273000	0.573688000
7	-7.639678000	0.821885000	1.633278000

Cartesian coordinates of BCL-10

6	3.586641000	-0.450305000	0.274875000
6	2.527250000	0.425317000	0.015697000
6	2.799416000	1.730507000	-0.396735000
6	4.113659000	2.136712000	-0.542354000
6	5.175331000	1.270157000	-0.286433000
6	4.892758000	-0.038331000	0.127077000
6	6.508559000	1.750940000	-0.455565000
6	6.020300000	-1.000029000	0.412491000
6	0.053378000	0.566131000	0.007361000
6	-1.168002000	-0.317184000	0.333242000
6	-1.924495000	0.359368000	1.474184000
6	-1.962173000	-0.378981000	-0.979692000
6	-4.741655000	-0.338940000	-0.296147000
6	-5.290475000	-0.622510000	0.944844000
6	-6.307472000	0.179701000	1.437306000
6	-6.737120000	1.248372000	0.668384000
6	-6.203324000	1.535628000	-0.578953000
6	-5.196821000	0.721964000	-1.068941000
9	5.565899000	-2.203784000	0.795847000
9	6.814443000	-0.543697000	1.391403000
9	6.785859000	-1.189656000	-0.671373000
1	-4.780694000	0.900060000	-2.052586000
1	-6.585381000	2.373915000	-1.145792000
1	-6.767943000	-0.010096000	2.397545000
1	-4.929747000	-1.472687000	1.508811000
1	-1.372830000	-0.872454000	-1.755403000
1	-2.233125000	0.616259000	-1.326948000
1	-2.779135000	-0.244874000	1.770609000
1	-1.263839000	0.456834000	2.335549000
1	-2.266935000	1.349827000	1.178679000
1	-1.477016000	-2.202095000	0.670311000
1	1.986235000	2.409762000	-0.598077000
1	3.383659000	-1.463768000	0.595630000
1	4.330101000	3.147646000	-0.861724000
1	1.139457000	-1.032341000	0.488238000
9	-7.711742000	2.031228000	1.143361000
16	-3.459978000	-1.377005000	-0.944179000
8	-3.781644000	-1.679775000	-2.319441000
8	-3.239370000	-2.452749000	0.019006000

8	-0.714463000	-1.604297000	0.707959000
8	-0.095691000	1.712507000	-0.367839000
7	1.240358000	-0.070672000	0.190673000
7	7.568431000	2.178200000	-0.603880000

Cartesian coordinates of JAYCES01

6	10.753600000	12.755800000	4.311000000
6	11.665900000	11.764000000	3.999700000
6	12.892000000	12.100700000	3.469300000
6	13.177600000	13.429200000	3.151900000
6	12.232200000	14.429600000	3.400400000
6	11.027300000	14.072000000	3.998700000
6	12.521900000	15.788600000	2.992700000
6	10.010900000	15.122600000	4.312100000
6	11.753600000	9.312800000	3.704600000
6	11.080000000	8.002700000	4.181200000
6	12.098000000	7.235000000	4.995600000
6	10.623300000	7.195800000	3.009900000
6	7.777000000	7.477400000	2.654000000
6	7.130300000	6.333800000	2.202700000
6	5.891800000	6.003200000	2.711800000
6	5.347100000	6.821100000	3.658000000
6	5.957800000	7.953700000	4.126500000
6	7.186800000	8.290400000	3.608300000
9	10.509100000	16.070300000	5.124400000
9	9.571900000	15.767800000	3.245200000
9	8.955500000	14.649900000	4.940800000
1	7.530800000	5.792600000	1.560700000
1	5.440600000	5.244100000	2.418700000
1	5.552500000	8.481400000	4.775500000
1	7.619500000	9.061800000	3.897300000
1	10.297600000	6.343600000	3.337500000
1	11.393000000	7.014600000	2.447100000
1	12.433000000	7.795800000	5.698400000
1	12.824100000	6.965600000	4.427700000
1	11.683600000	6.458700000	5.375900000
1	9.533900000	8.961400000	4.661900000
1	13.531900000	11.440800000	3.322300000
1	9.955000000	12.535400000	4.731900000
1	13.999200000	13.650800000	2.774600000
1	10.730900000	10.327800000	4.941800000
9	4.130800000	6.497900000	4.168100000
16	9.343700000	7.928700000	1.988600000
8	9.483700000	9.353200000	2.052600000
8	9.467400000	7.286400000	0.699700000
8	9.934500000	8.327100000	4.993600000
8	12.634200000	9.276100000	2.873000000
7	11.300800000	10.434300000	4.306000000
7	12.745500000	16.836700000	2.642800000

Cartesian coordinates of JAYCES02

6	8.968600000	7.712300000	2.573100000
6	9.029400000	8.712000000	1.609800000

6	9.743000000	8.485200000	0.435800000
6	10.362400000	7.272800000	0.226200000
6	10.283200000	6.262600000	1.167400000
6	9.572100000	6.479500000	2.351300000
6	10.953700000	5.017100000	0.925700000
6	9.399000000	5.401000000	3.367500000
6	7.896300000	10.474600000	2.930500000
6	7.274000000	11.881800000	2.795900000
6	8.164500000	12.844500000	3.541800000
6	5.902500000	11.942500000	3.395100000
6	4.846300000	9.323200000	3.097200000
6	4.611300000	8.888200000	4.392500000
6	4.878000000	7.591300000	4.729000000
6	5.353000000	6.748700000	3.782300000
6	5.580300000	7.136600000	2.520100000
6	5.327300000	8.443700000	2.156000000
9	10.572800000	4.850300000	3.718400000
9	8.666300000	4.404400000	2.922800000
9	8.867100000	5.819000000	4.491800000
1	4.272800000	9.475100000	5.029200000
1	4.735500000	7.291800000	5.597400000
1	5.904600000	6.530000000	1.894500000
1	5.481000000	8.727000000	1.283200000
1	5.633100000	12.873800000	3.397300000
1	5.985100000	11.672400000	4.323000000
1	9.048600000	12.819100000	3.167800000
1	8.201700000	12.595000000	4.467500000
1	7.810400000	13.733800000	3.464600000
1	6.935300000	12.982100000	1.293100000
1	9.801900000	9.154200000	-0.207400000
1	8.519100000	7.868300000	3.371900000
1	10.839300000	7.131700000	-0.559400000
1	8.282300000	10.426300000	1.075800000
9	5.602800000	5.466900000	4.120000000
16	4.540600000	11.000700000	2.688600000
8	3.398700000	11.446700000	3.419300000
8	4.561600000	11.126300000	1.271100000
8	7.282800000	12.245000000	1.388000000
8	7.954400000	9.933700000	4.017400000
7	8.382000000	9.948800000	1.784100000
7	11.498700000	4.036000000	0.729300000

Cartesian coordinates of FAHFIG

6	-2.674200000	2.627000000	15.522400000
6	-1.760000000	3.628100000	15.163100000
6	-1.795100000	4.849000000	15.809600000
6	-2.738300000	5.099600000	16.783900000
6	-3.668200000	4.118600000	17.128900000
6	-3.618200000	2.881100000	16.496000000
6	-4.668100000	4.460600000	18.125000000
6	-4.576000000	1.792100000	16.872400000
6	-0.608400000	2.394200000	13.343700000
6	0.385100000	2.617500000	12.199100000

6	-0.456800000	2.679200000	10.915000000
6	1.363200000	1.447700000	12.102500000
6	3.798400000	2.432200000	13.183500000
6	4.755500000	2.154300000	12.237400000
6	5.783000000	3.028400000	12.031600000
6	5.824300000	4.162500000	12.794900000
6	4.888300000	4.471300000	13.737900000
6	3.843700000	3.596100000	13.931600000
9	-4.609000000	0.794500000	16.016300000
9	-5.840500000	2.230300000	16.900600000
9	-4.329600000	1.298000000	18.040200000
1	3.178900000	3.783700000	14.555400000
1	4.953500000	5.253900000	14.236500000
1	6.436200000	2.857400000	11.393900000
1	4.701800000	1.371700000	11.737800000
1	1.878900000	1.548600000	11.287600000
1	0.849900000	0.628200000	12.021900000
1	-1.071200000	3.414400000	10.970600000
1	-0.945500000	1.859800000	10.814100000
1	0.121200000	2.802700000	10.159000000
1	1.495900000	3.966600000	11.791900000
1	-1.178100000	5.508100000	15.585400000
1	-2.644800000	1.795700000	15.106100000
1	-2.752700000	5.923700000	17.212500000
1	-0.319400000	4.109100000	13.993000000
9	6.838900000	5.044900000	12.597000000
16	2.495200000	1.269100000	13.463300000
8	1.810400000	1.616700000	14.677100000
8	3.068000000	-0.046100000	13.347200000
8	1.068600000	3.843100000	12.431000000
8	-1.191400000	1.331300000	13.448300000
7	-0.803700000	3.463000000	14.148800000
7	-5.427700000	4.780100000	18.905000000

Cartesian coordinates of KIHZOR

6	4.430600000	4.600300000	6.802800000
6	4.056200000	5.635800000	5.940600000
6	2.753000000	6.018800000	5.890100000
6	1.798300000	5.392100000	6.664400000
6	2.150000000	4.373300000	7.535300000
6	3.484200000	3.965100000	7.588700000
6	1.093500000	3.817300000	8.341300000
6	3.943200000	2.868900000	8.446600000
6	6.338900000	6.225200000	5.151900000
6	7.066400000	7.084000000	4.080600000
6	7.780100000	8.224600000	4.857700000
6	8.129100000	6.203900000	3.388400000
6	6.136300000	4.355400000	2.575200000
6	4.939100000	4.759400000	2.015800000
6	3.757400000	4.150900000	2.409400000
6	3.823100000	3.158700000	3.366800000
6	4.994600000	2.741900000	3.929200000
6	6.179300000	3.339800000	3.519700000

9	4.054600000	3.171100000	9.649100000
9	4.978800000	2.273300000	8.078900000
9	3.070600000	1.895300000	8.550400000
1	6.992500000	3.063400000	3.875800000
1	4.999200000	2.070000000	4.572200000
1	2.943100000	4.403200000	2.037400000
1	4.926000000	5.437200000	1.378400000
1	8.537300000	5.646100000	4.070500000
1	8.821900000	6.791800000	3.049600000
1	8.287800000	8.788800000	4.216100000
1	6.828600000	8.769700000	5.053800000
1	8.432500000	7.621300000	5.459000000
1	6.573900000	8.198000000	2.374800000
1	2.501300000	6.712900000	5.324900000
1	5.321100000	4.335300000	6.847500000
1	0.908500000	5.655900000	6.601000000
1	4.618200000	6.871300000	4.364600000
9	2.670900000	2.589600000	3.764800000
16	7.627600000	5.143700000	2.058400000
8	7.347900000	5.931600000	0.899700000
8	8.650800000	4.129900000	1.959500000
8	6.120100000	7.574700000	3.172200000
8	6.966400000	5.583200000	5.983900000
7	4.989000000	6.309000000	5.107200000
7	0.223100000	3.456600000	8.981500000

Cartesian coordinates of KIHZIL

6	7.046400000	7.681600000	7.794900000
6	6.576100000	8.486600000	6.750800000
6	5.229400000	8.825000000	6.724200000
6	4.370300000	8.380600000	7.695000000
6	4.823200000	7.594800000	8.739500000
6	6.182000000	7.248700000	8.769000000
6	3.909200000	7.180800000	9.768100000
6	6.717000000	6.407900000	9.893700000
6	8.735700000	8.935000000	5.654100000
6	9.326500000	9.631900000	4.431000000
6	9.520500000	11.098600000	4.807500000
6	10.686200000	9.010600000	4.109800000
6	11.982600000	6.538300000	4.208200000
6	11.799500000	5.755700000	5.317500000
6	12.899400000	5.128600000	5.892200000
6	14.133200000	5.342400000	5.336200000
6	14.335900000	6.116600000	4.258000000
6	13.247900000	6.719800000	3.672900000
9	7.993700000	6.129100000	9.769900000
9	6.081000000	5.257300000	10.006100000
9	6.569200000	7.006400000	11.068600000
1	13.361400000	7.250300000	2.916600000
1	15.191300000	6.241500000	3.914300000
1	12.800000000	4.578300000	6.634300000
1	10.951200000	5.645700000	5.682100000
1	11.219900000	8.999300000	4.919300000

1	11.140900000	9.576800000	3.466400000
1	10.055100000	11.188100000	5.549200000
1	9.927200000	11.556800000	4.056300000
1	8.645000000	11.549500000	5.059500000
1	8.686700000	10.187100000	2.750900000
1	4.905600000	9.361500000	6.037100000
1	7.944200000	7.441200000	7.828600000
1	3.470600000	8.610700000	7.650200000
1	7.015100000	9.366400000	4.975700000
9	15.193300000	4.733600000	5.912100000
16	10.608200000	7.356600000	3.456200000
8	9.413400000	6.708300000	3.907800000
8	10.857400000	7.419000000	2.043800000
8	8.447900000	9.496200000	3.327500000
8	9.454600000	8.483600000	6.522300000
7	7.379500000	8.954700000	5.714400000
7	3.180400000	6.884500000	10.586100000

Cartesian coordinates of 1Z95

6	26.365600000	3.087900000	4.819000000
6	27.705600000	3.321900000	4.518000000
6	28.094600000	4.602900000	4.142000000
6	27.149600000	5.621900000	4.083000000
6	25.814600000	5.372900000	4.388000000
6	25.394600000	4.089900000	4.755000000
6	24.932700000	6.498800000	4.324000000
6	23.930700000	3.730900000	5.075000000
6	28.448600000	1.087000000	4.823000000
6	29.680600000	0.203000000	4.612000000
6	29.275600000	-1.021000000	3.788000000
6	30.256600000	-0.233000000	5.962000000
6	30.415600000	0.948000000	8.622900000
6	31.209600000	0.269000000	9.540900000
6	30.772600000	0.107000000	10.851900000
6	29.542600000	0.626000000	11.243900000
6	28.747600000	1.307000000	10.326900000
6	29.184600000	1.467000000	9.014900000
9	23.713700000	3.871900000	6.379000000
9	23.029700000	4.472900000	4.437000000
9	23.639700000	2.473900000	4.749000000
1	28.574400000	1.988900000	8.307700000
1	27.802400000	1.706800000	10.629700000
1	31.382200000	-0.416500000	11.558400000
1	32.156000000	-0.128600000	9.239100000
1	29.425700000	-0.607700000	6.522400000
1	31.079300000	-0.876600000	5.730000000
1	30.086800000	-1.306700000	3.151400000
1	29.036200000	-1.830400000	4.445600000
1	28.420600000	-0.781900000	3.190700000
1	30.338300000	1.167100000	3.024800000
1	29.117000000	4.804000000	3.899000000
1	26.068700000	2.102100000	5.110600000
1	27.452400000	6.608400000	3.799900000

1	29.530415209	2.611234398	4.127159594
9	29.126600000	0.466000000	12.515900000
16	30.960600000	1.123000000	6.971000000
8	30.500600000	2.449900000	6.443000000
8	32.457500000	1.053000000	6.927000000
8	30.665600000	0.959000000	3.903000000
8	27.443600000	0.641000000	5.376000000
7	28.624600000	2.356900000	4.466000000
7	24.135800000	7.516100000	4.266200000

Cartesian coordinates of JAYCES

6	1.576000000	6.644400000	5.836800000
6	0.653700000	5.643100000	6.130500000
6	-0.567500000	5.983500000	6.676300000
6	-0.838200000	7.304400000	6.992600000
6	0.093500000	8.295200000	6.751000000
6	1.300700000	7.956800000	6.144400000
6	-0.193200000	9.649100000	7.168700000
6	2.306600000	9.012200000	5.824800000
6	0.571400000	3.194400000	6.421900000
6	1.242000000	1.899900000	5.933900000
6	0.221900000	1.116600000	5.127600000
6	1.682800000	1.069200000	7.130700000
6	4.507400000	1.349000000	7.476200000
6	5.145200000	0.207000000	7.930700000
6	6.376300000	-0.124700000	7.410500000
6	6.927000000	0.680500000	6.475500000
6	6.323500000	1.816800000	6.009000000
6	5.093600000	2.159700000	6.519100000
9	3.363600000	8.532100000	5.205500000
9	2.736000000	9.657100000	6.895900000
9	1.804000000	9.962000000	5.038200000
1	4.651000000	2.922600000	6.224500000
1	6.738900000	2.338800000	5.361300000
1	6.830500000	-0.885400000	7.692900000
1	4.737100000	-0.322400000	8.577300000
1	0.904400000	0.914800000	7.687800000
1	2.016400000	0.221100000	6.800300000
1	0.013100000	1.597500000	4.324100000
1	-0.576500000	1.003900000	5.648700000
1	0.580600000	0.255300000	4.901900000
1	2.210000000	2.034700000	4.294700000
1	-1.204600000	5.323600000	6.830700000
1	2.383700000	6.422800000	5.432200000
1	-1.656200000	7.529300000	7.373100000
1	1.705200000	4.236700000	5.352200000
9	8.142500000	0.353600000	5.970000000
16	2.949000000	1.796300000	8.148500000
8	2.821000000	3.226800000	8.073600000
8	2.821100000	1.162300000	9.438300000
8	2.385500000	2.227500000	5.165300000
8	-0.305700000	3.156800000	7.261800000
7	1.016800000	4.325500000	5.822400000

7 -0.422000000 10.704300000 7.518100000

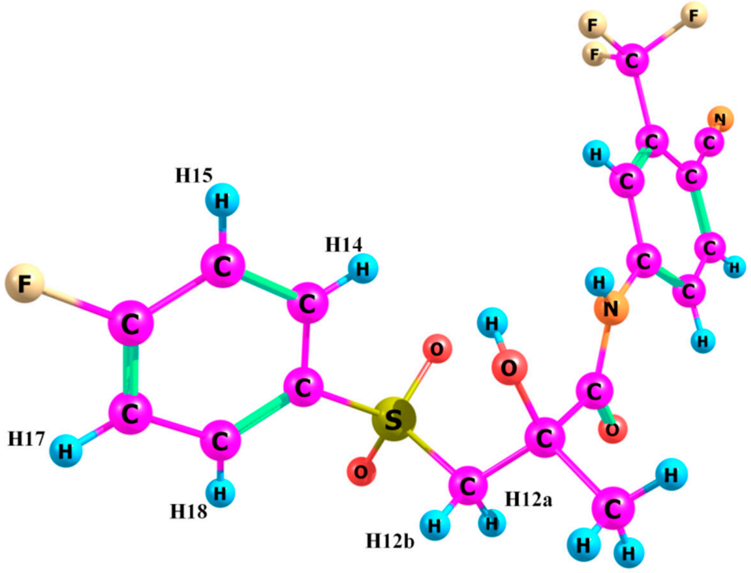

JAYCES01 - Open
$r_{H12b-H14/18} = 3.53$ Å
$r_{H12a-H12b} = 1.56$ Å
$r_{H15/17-H14/18} = 2.60$ Å

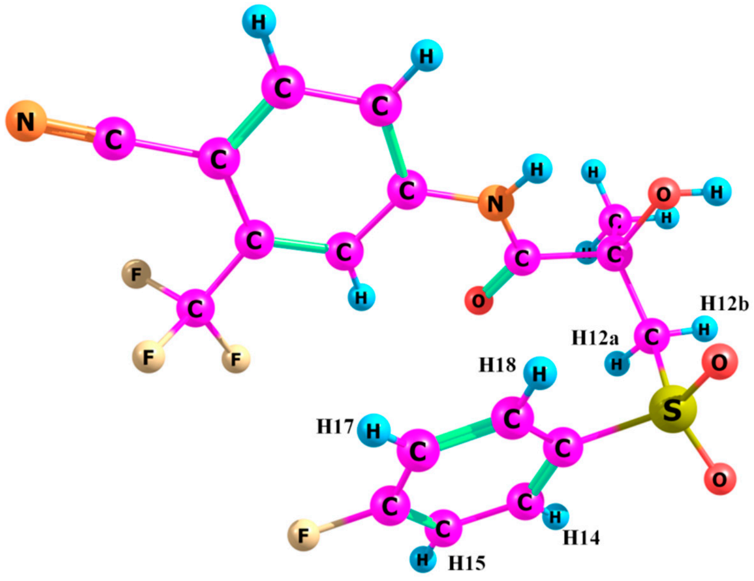

JAYCES02 - Close
$r_{H12b-H14/18} = 4.25$ Å
$r_{H12a-H12b} = 1.56$ Å
$r_{H15/17-H14/18} = 2.59$ Å

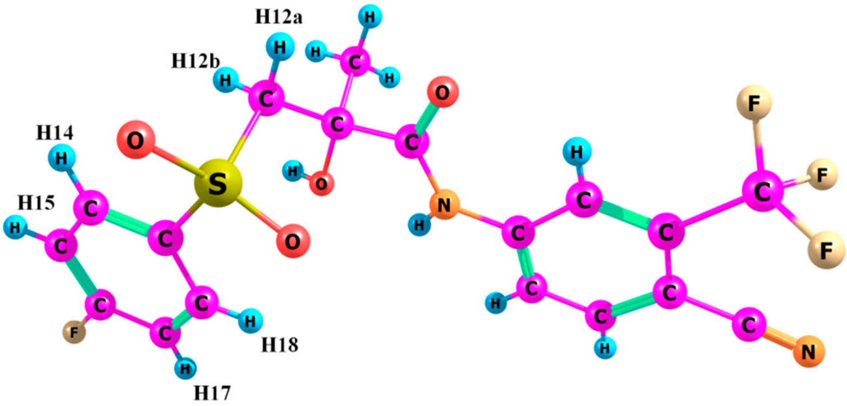

FAHFIG - Open
$r_{H12b-H14/18} = 3.16$ Å
$r_{H12a-H12b} = 1.56$ Å
$r_{H15/17-H14/18} = 2.59$ Å

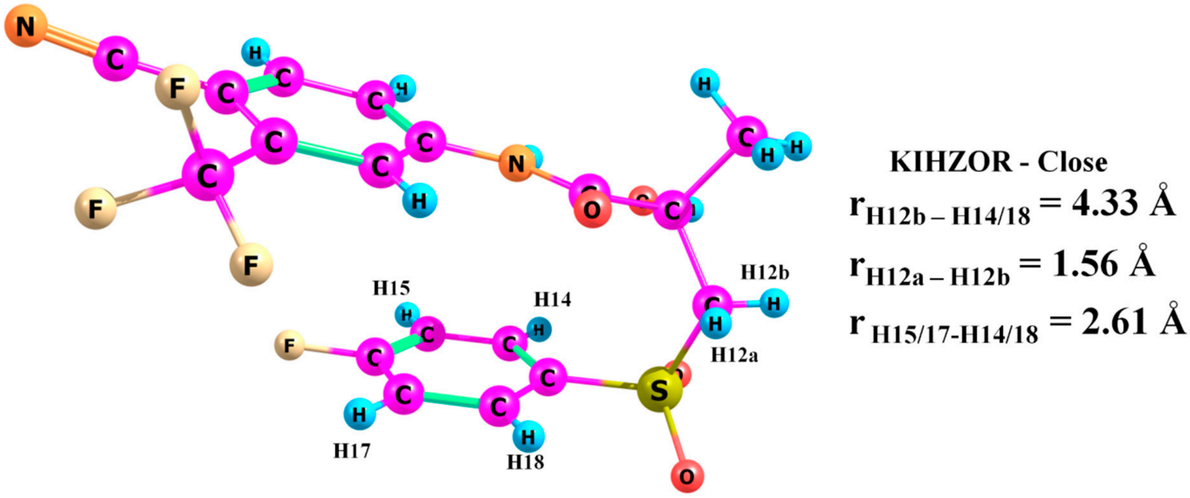
H15
H14
H12b
H12a
H17
H18
KIHZOR - Close
$r_{H12b-H14/18}$ = 4.33 Å
$r_{H12a-H12b}$ = 1.56 Å
$r_{H15/17-H14/18}$ = 2.61 Å

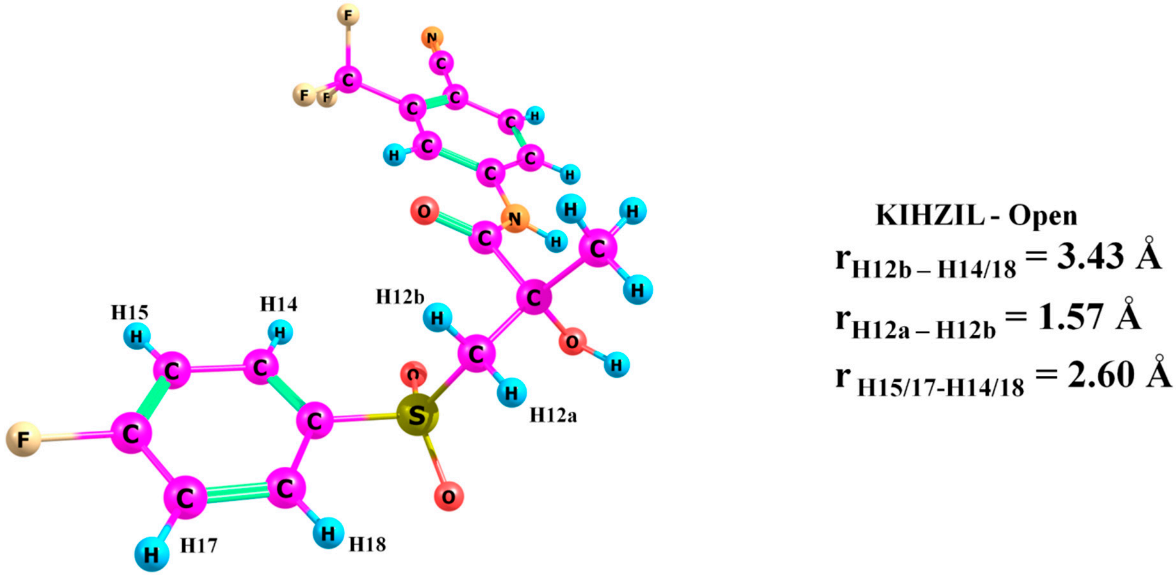
H15
H14
H12b
H12a
H17
H18
KIHZIL - Open
$r_{H12b-H14/18}$ = 3.43 Å
$r_{H12a-H12b}$ = 1.57 Å
$r_{H15/17-H14/18}$ = 2.60 Å

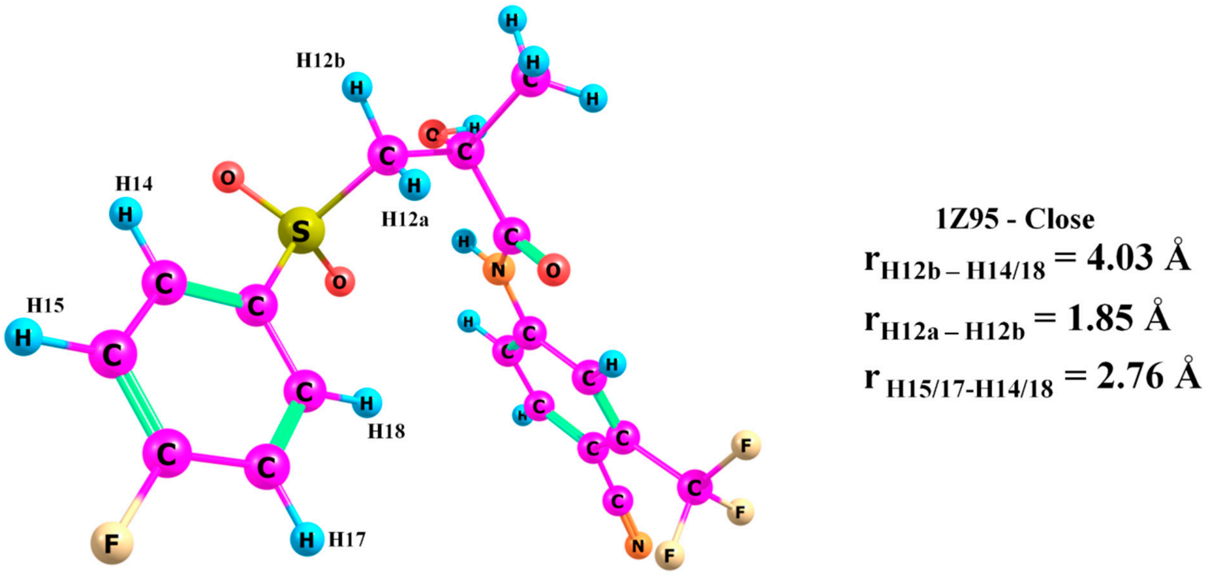
H12b
H14
H12a
H15
H18
H17
1Z95 - Close
$r_{H12b-H14/18}$ = 4.03 Å
$r_{H12a-H12b}$ = 1.85 Å
$r_{H15/17-H14/18}$ = 2.76 Å

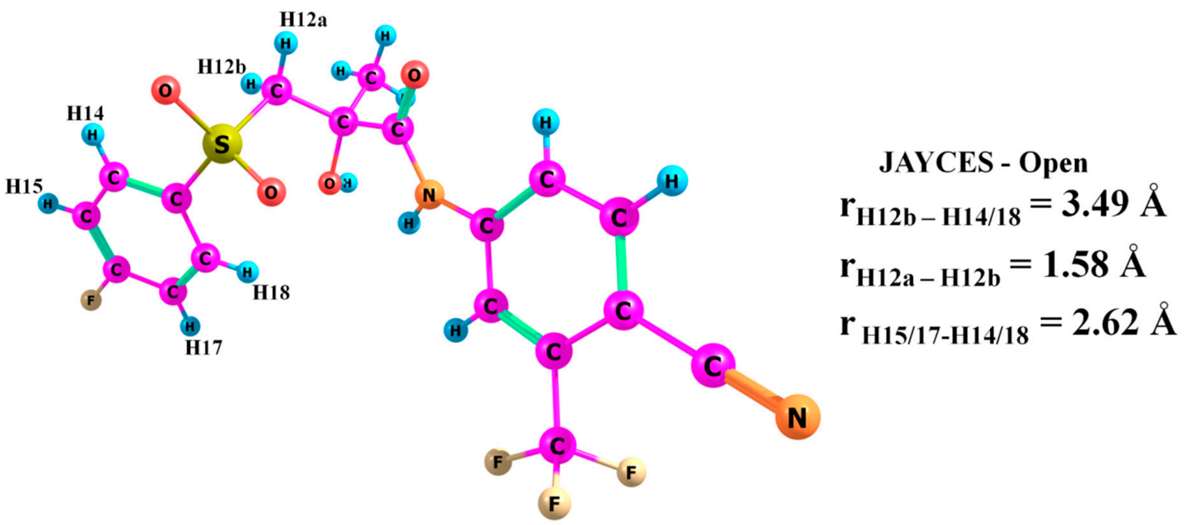

Figure S5. BCL Conformers obtained from CCDC and PDB data, including CSD Refcode and PDB ID, as well as the distances H12b-H14/18, H12a-H12b, and H15/17-H14/18, utilized for calculating conformer group proportions

Table S1. Spin-Spin Coupling Constants, ^{1}H and ^{13}C Chemical Shifts, and Cross-Correlation Peaks in 2D Spectra of Bicalutamide in $CDCl_3$

δ ^{13}C	^{1}H	δ ^{1}H	J, Hz	HMBC	HSQC	TOCSY		NOESY	^{13}C
						20 ms	100 ms		
116.80	H1	8.01	s	C1-NH	C1-H1	H1-H3/4	H1-H3/4	H1-NH	C1
141.00	-	-		C2-H1<br>C2-H3/4	-	-			C2
121.50	H3/4	7.83	-	C3-NH	C3-H3	-		H3/4-NH	C3
136.20					C4-H4	-			C4
116.35	-	-			-	-			C5
140.60	-	-			-	-			C6
104.90	-	-		C7-H1<br>C7-H3/4	-	-			C7
122.98	-	-			-	-			C8
171.40	-	-		C9-OH<br>C9-H12a<br>C9-H11	-	-			C9
74.30	-	-		C10-<br>H12a<br>C10-<br>H12b<br>C10-H11<br>C10-OH	-	-			C10
27.40	H11	1.62	s	C11-OH	C11-H11	-		H11-NH<br>H11-<br>H12a<br>H11-<br>H12b	C11
62.50	H12a	3.51	d,	C12-H11	C12-	-	H12a-	H12a-	C12

δ ^{13}C	^{1}H	δ ^{1}H	J, Hz	HMBC	HSQC	TOCSY		NOESY	^{13}C
						20 ms	100 ms		
			14.45		C12a		H12b H12a-H11	H14/18 H12a-H12b H12a-OH	
	H12b	3.96	d, 14.45		C12-C12b	H12b-H12a	H12b-H11	H12b-H14/18	
165.40	-	-		C13-H14/18 C13-H15/17	-	-			C13
131.70	H14/H18	7.91	m, 13.50; 3.75		C14/18-H14/18	-		H14/18-H15/17	C14/C18
116.40	H15/17	7.21	t, 8.38		C15/17-H15/17	H14/18-H15/17	H14/18-H15/17		C15/C17
166.30	-	-		C16-H14/18 C16-H15/17	-	-			C16
-	NH	9.09	s		-	-	NH-OH NH-H11	NH-OH	-
-	OH	5.07	s		-	OH-H11	OH-H12b OH-H12a		-

Table S2. Spin-Spin Coupling Constants,^{1}H and ^{13}C, Chemical Shifts, and Cross-Correlational Peaks in the 2D Spectra of BCL in DMSO-d6

δ ^{13}C	^{1}H	δ ^{1}H	J, Hz	HMBC	HSQC	TOCSY		NOESY	^{13}C
						20 ms	100 ms		
117.95	H1	8.45	d, 1.15	C1-NH	C1-H1	H1-H3	H1-H3<br>H1-H4<br>H1-H11		C1
143.63	-	-		C2-NH<br>C2-H3<br>C2-H1<br>C2-H4	-				C2
123.30	H3	8.24	dd, 8.60; 1.35	C3-NH<br>C3-H4<br>C3-H1	C3-H3	H3-H4		H3-NH<br>H3-H4	C3
136.60	H4	8.09	d, 8.60	C4-NH<br>C4-H3	C4-H4				C4
116.34	-	-		C5-H4	-				C5
137.59	-	-		-	-				C6
102.42	-	-		C7-H1<br>C7-H3	-				C7
122.97	-	-		-	-				C8
174.21	-	-		C9-NH<br>C9-OH<br>C9-H12a<br>C9-H12b<br>C9-H11	-				C9
73.59	-	-		C10-OH<br>C10-H12a<br>C10-H12b<br>C10-H11	-				C10
27.64	H11	1.43	s	C11-OH<br>C11-H12a<br>C11-H12b	C11-H11			H11-NH<br>H11-H14/18<br>H11-OH<br>H11-H12b<br>H11-H12a	C11
63.94	H12a	3.74	d, 14.86	C12-OH<br>C12-H11	C12-H12a	H12a-H12b	H12a-H12b<br>H12a-H11	H12a-H14/18<br>H12a-OH<br>H12a-H12b	C12
	H12b	3.97	d, 14.86		C12-H12b		H12b-H11	H12b-H14/18<br>H12b-	

δ ^{13}C	^{1}H	δ ^{1}H	J, Hz	HMBC	HSQC	TOCSY		NOESY	^{13}C
						20 ms	100 ms		
								OH	
164.33	-	-		C13-H14/18 C13-H15/17	-				C13
131.85	H14/H18	7.94	m, 13.90; 3.50	-	C14/18-H14/18	H14/18-H15/17	H14/18-H15/17 H14/18-H12a H14/18-H12b	H14/18-NH	C14/C18
116.59	H15/17	7.37	t, 8.78	C15/17-H14/18	C15/17-H15/17		H15/17-OH H15/17-H12b H15/17-H12a	H15/17-H14/18	C15/C17
166.34	-	-		C16-H14/18 C16-H15/17	-				C16
-	NH	10.41	s	-	-		NH-H1 NH-H3 NH-H4 NH-H12a NH-H12b NH-H11		-
-	OH	6.44	s	-	-		OH-NH OH-H12a OH-H12b OH-H11	OH-H14/18	-

Figure S6. Average integral intensity of distances derived from NOESY spectral analysis for bicalutamide in $CDCl_3$

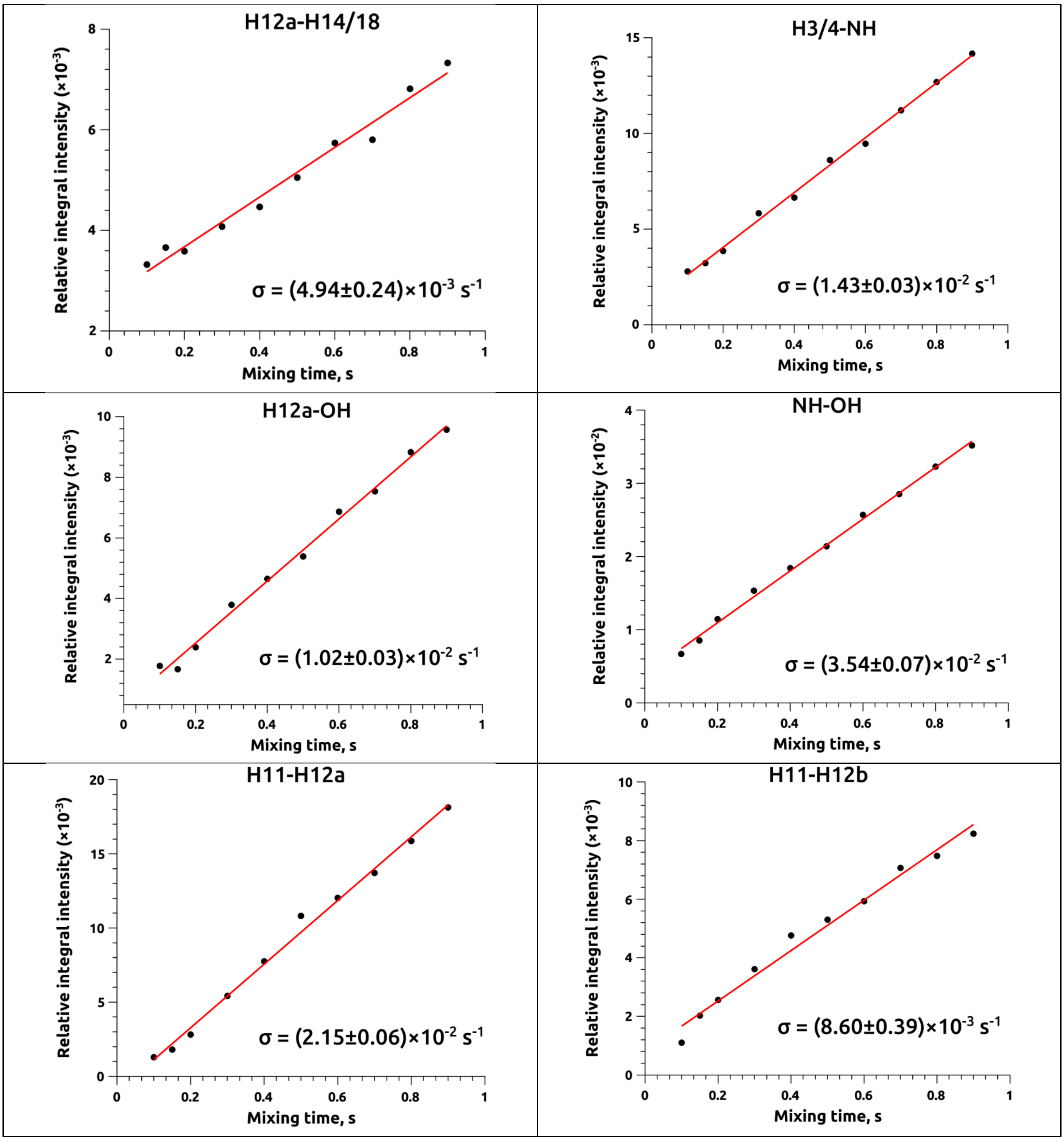

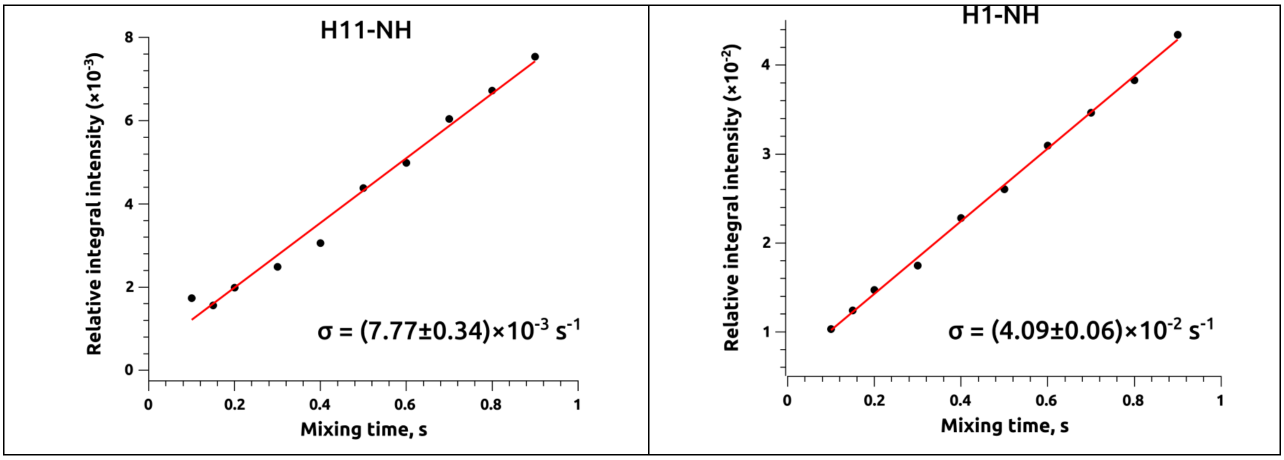

Figure S7. Average integral intensity of distances derived from NOESY spectral analysis for bicalutamide in DMSO-d_6

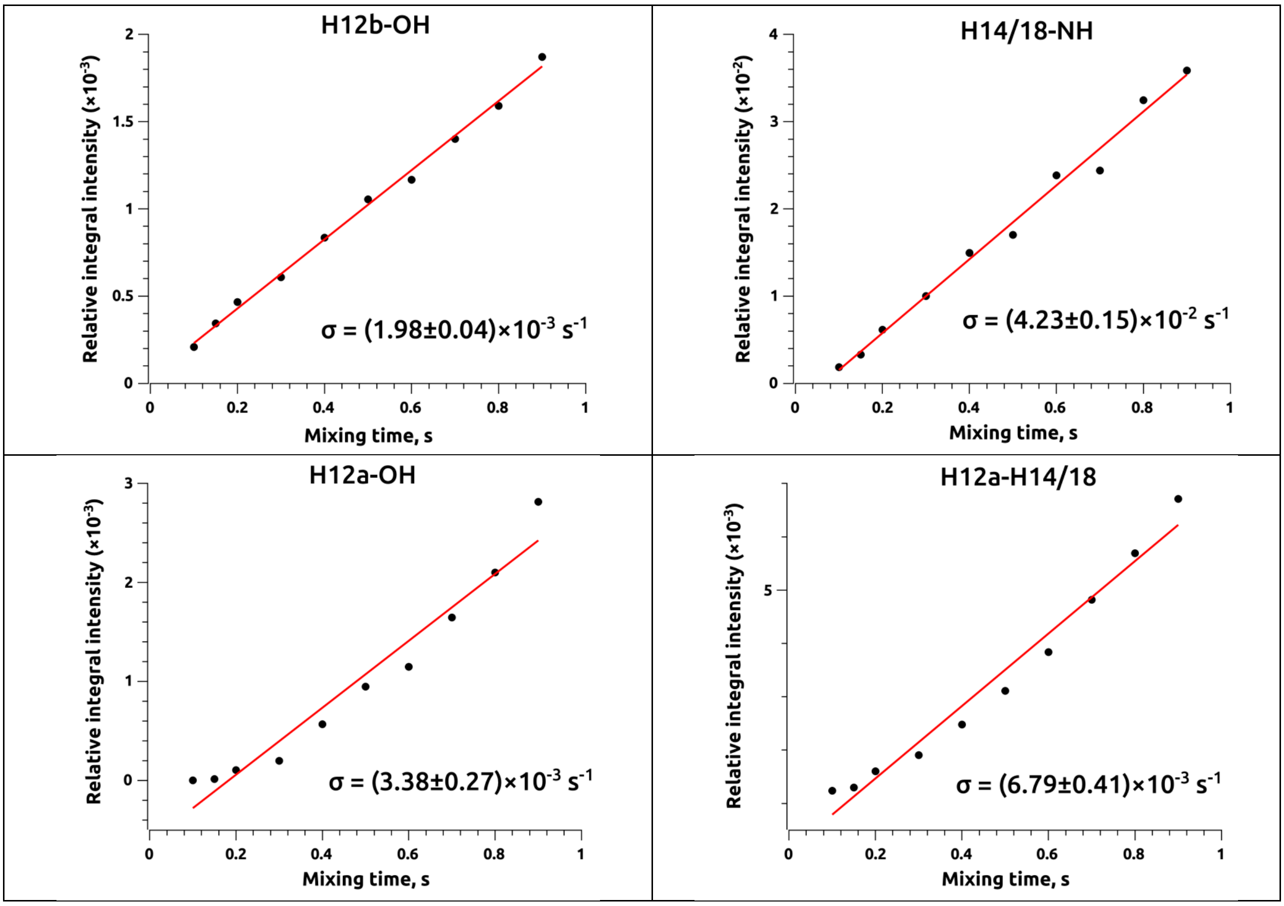

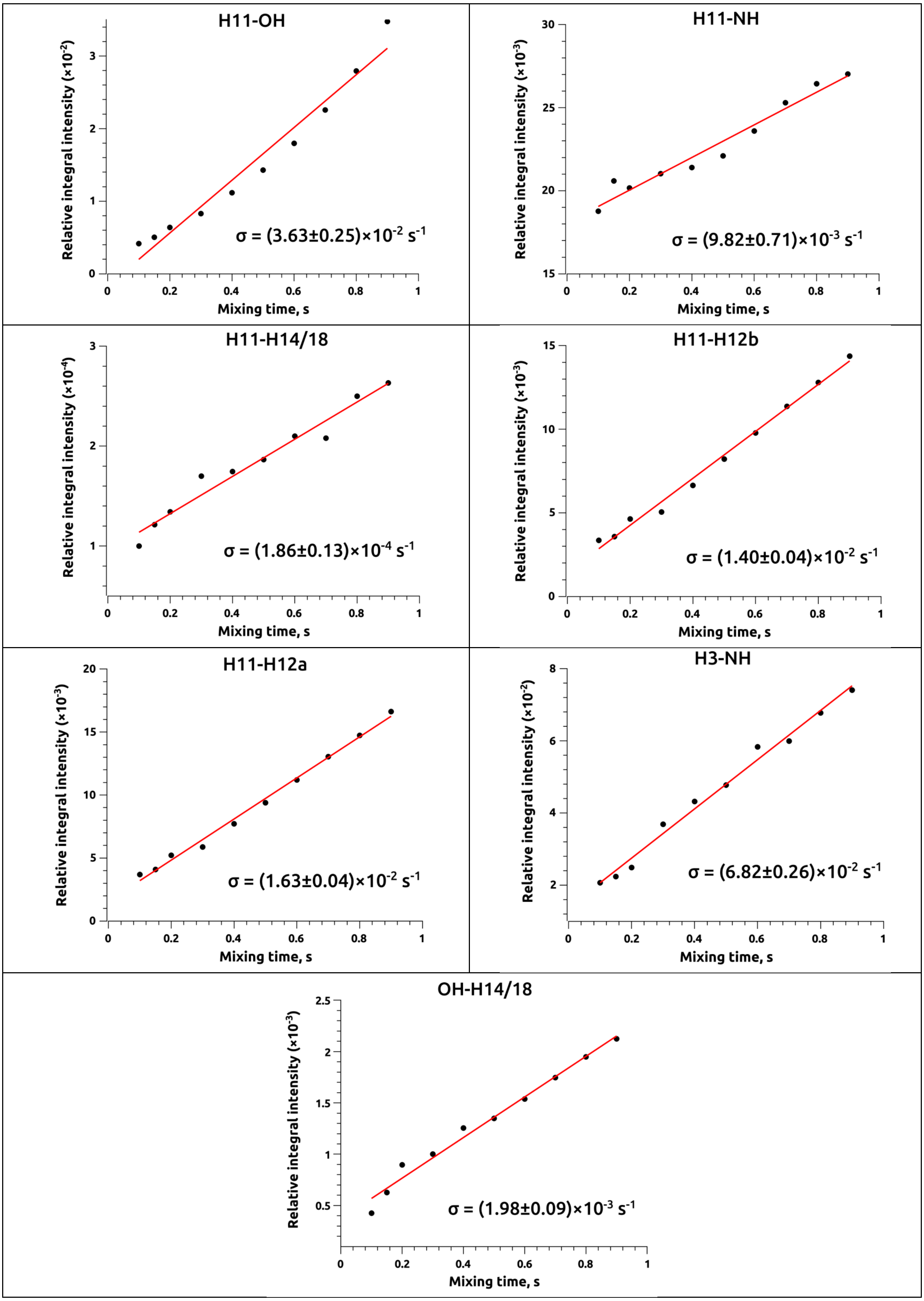
H11-OH
Relative integral intensity (×10⁻²)
σ = (3.63±0.25)×10⁻² s⁻¹
Mixing time, s
H11-NH
Relative integral intensity (×10⁻³)
σ = (9.82±0.71)×10⁻³ s⁻¹
Mixing time, s
H11-H14/18
Relative integral intensity (×10⁻⁴)
σ = (1.86±0.13)×10⁻⁴ s⁻¹
Mixing time, s
H11-H12b
Relative integral intensity (×10⁻³)
σ = (1.40±0.04)×10⁻² s⁻¹
Mixing time, s
H11-H12a
Relative integral intensity (×10⁻³)
σ = (1.63±0.04)×10⁻² s⁻¹
Mixing time, s
H3-NH
Relative integral intensity (×10⁻²)
σ = (6.82±0.26)×10⁻² s⁻¹
Mixing time, s
OH-H14/18
Relative integral intensity (×10⁻³)
σ = (1.98±0.09)×10⁻³ s⁻¹
Mixing time, s

Figure S8. Dependence of the cross-relaxation rate on the internuclear distance for conformers for bicalutamide in $CDCl_3$

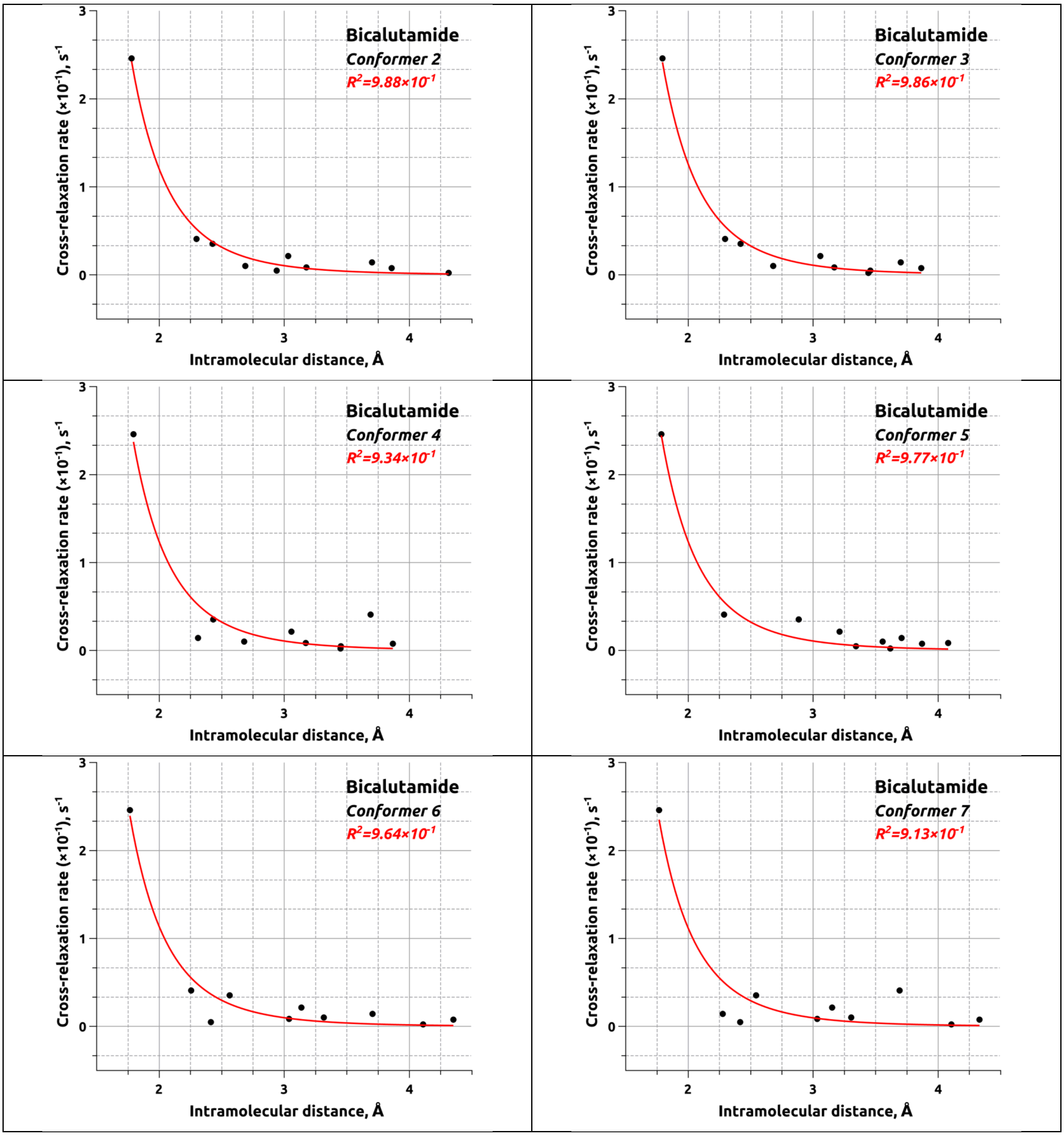

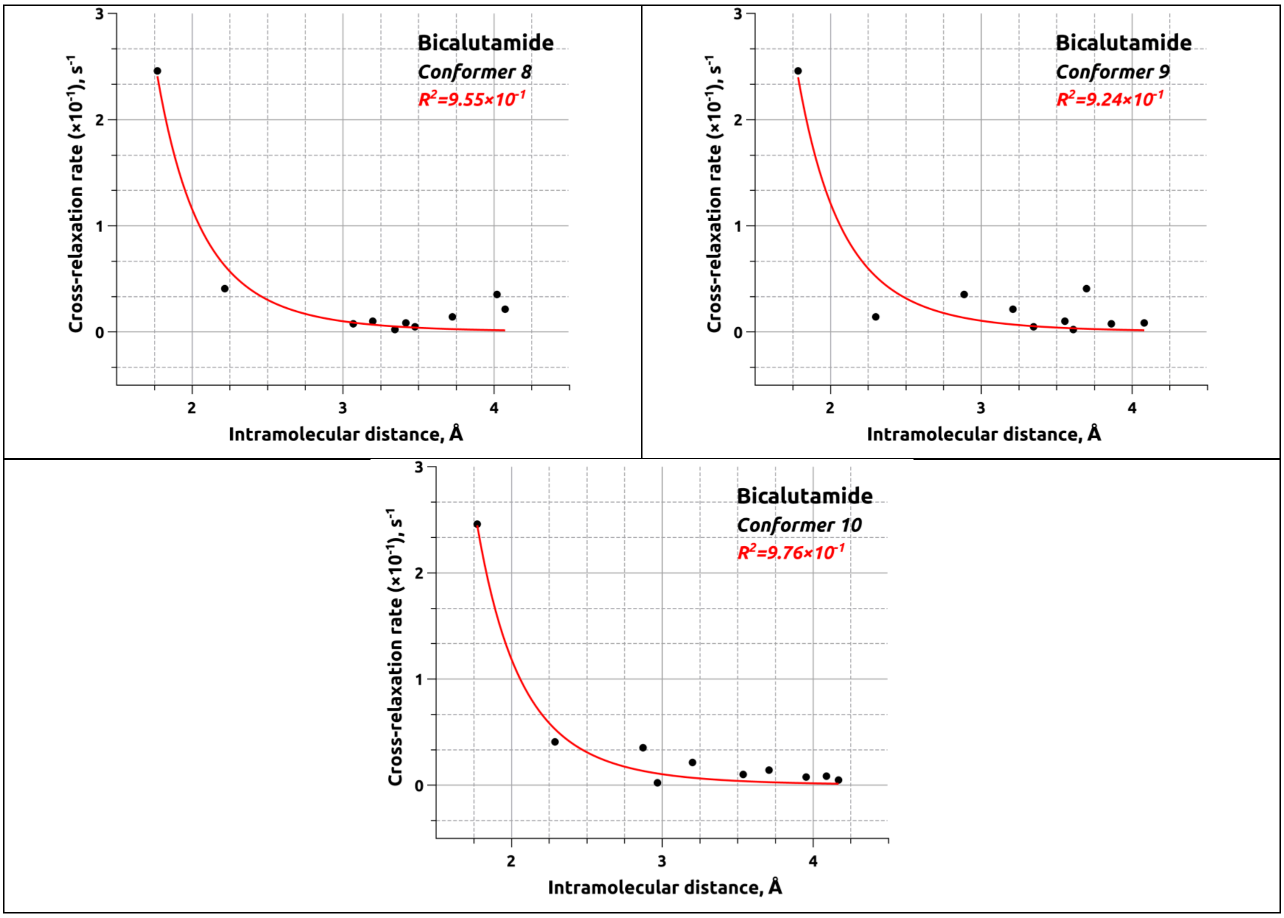

Figure S9. Dependence of the cross-relaxation rate on the internuclear distance for conformers for bicalutamide in DMSO-d6

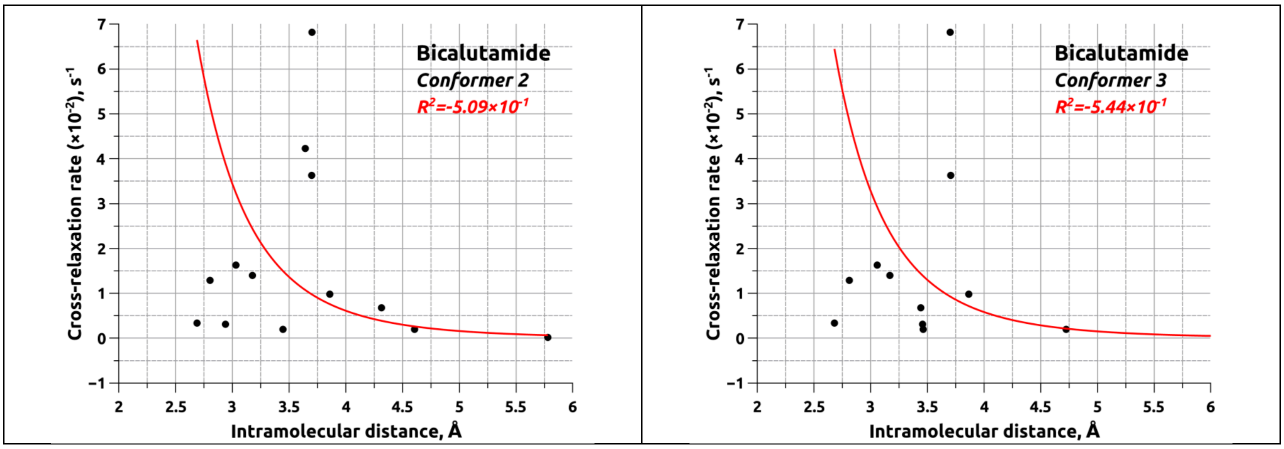

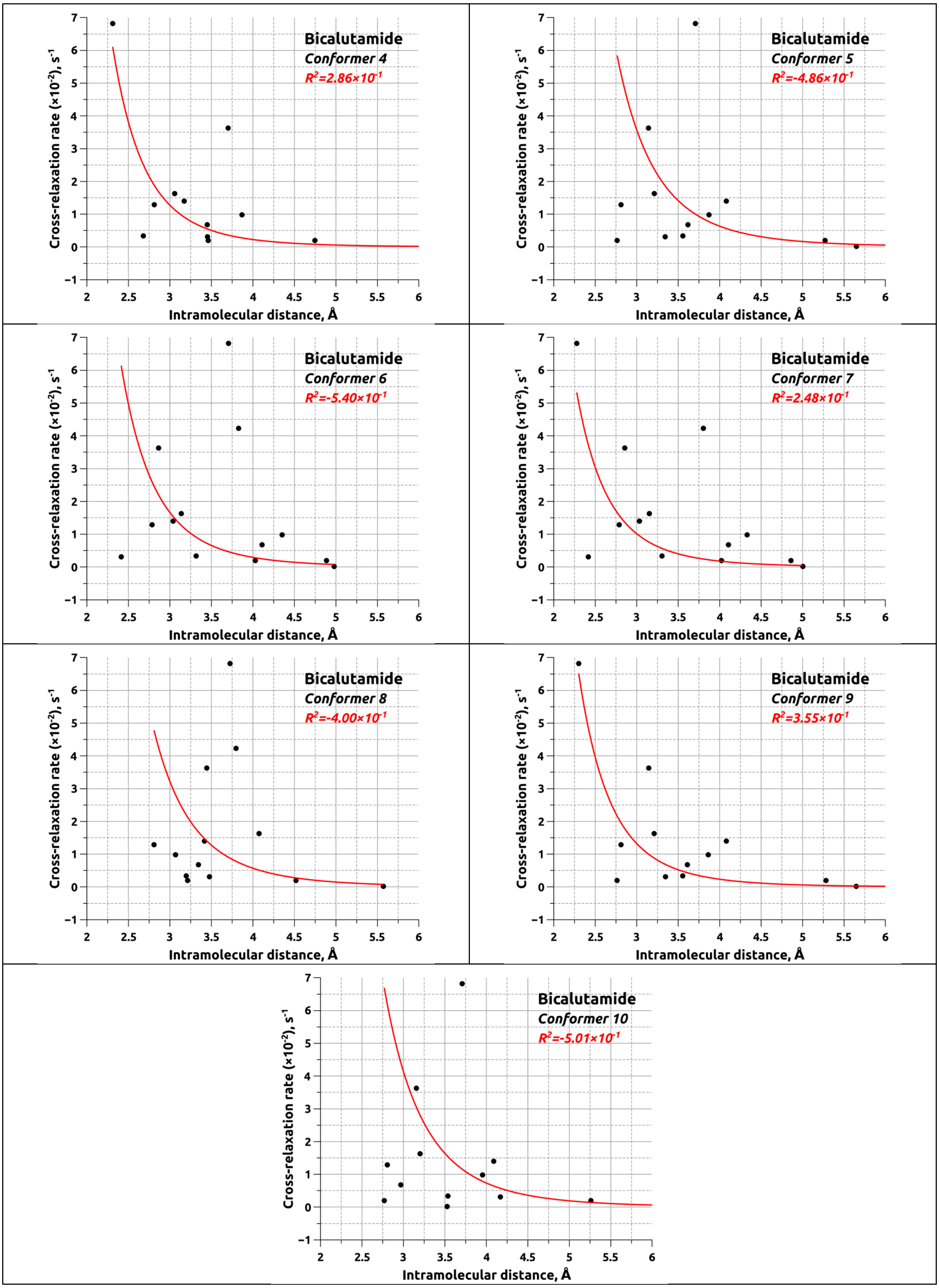
Bicalutamide
Conformer 4
R^2=2.86×10^{-1}
Bicalutamide
Conformer 5
R^2=-4.86×10^{-1}
Bicalutamide
Conformer 6
R^2=-5.40×10^{-1}
Bicalutamide
Conformer 7
R^2=2.48×10^{-1}
Bicalutamide
Conformer 8
R^2=-4.00×10^{-1}
Bicalutamide
Conformer 9
R^2=3.55×10^{-1}
Bicalutamide
Conformer 10
R^2=-5.01×10^{-1}
Cross-relaxation rate (×10^{-2}), s^{-1}
Intramolecular distance, Å

Table S3. RMSD Values (Å) Derived from the Comparison of BCL Conformer Structures Obtained from CCDC, PDB, and Quantum Chemical Calculations

	BCL-1	BCL-2	BCL-3	BCL-4	BCL-5	BCL-6	BCL-7	BCL-8	BCL-9	BCL-10	JAYCES01	JAYCES02	FAHFIG	KIHZOR	KIHZIL	1Z95	JAYCES
BCL-1	0.00	2.13	3.64	2.83	3.77	2.29	0.78	2.52	3.19	4.12	2.79	2.56	3.54	0.91	2.40	3.37	2.76
BCL-2	2.13	0.00	2.95	3.43	3.25	0.74	2.18	3.11	3.86	3.72	3.17	2.17	2.98	2.37	2.83	2.77	3.15
BCL-3	3.64	2.95	0.00	2.12	1.49	2.97	3.67	2.69	2.48	2.39	2.38	3.01	2.07	3.60	2.24	2.30	2.68
BCL-4	2.83	3.43	2.12	0.00	2.53	3.43	2.86	1.77	1.42	2.81	1.66	3.25	2.84	2.87	1.23	3.02	2.09
BCL-5	3.77	3.25	1.49	2.53	0.00	3.43	3.97	2.98	2.10	2.09	2.58	3.08	2.04	3.60	2.54	2.13	2.56
BCL-6	2.29	0.74	2.97	3.43	3.43	0.00	2.14	3.13	3.96	3.85	3.16	2.32	3.05	2.63	2.81	2.90	3.18
BCL-7	0.78	2.18	3.67	2.86	3.97	2.14	0.00	2.60	3.38	4.17	2.81	2.73	3.57	1.41	2.45	3.51	2.81
BCL-8	2.52	3.11	2.69	1.77	2.98	3.13	2.60	0.00	2.16	3.13	1.94	2.64	3.11	2.47	1.58	2.76	2.14
BCL-9	3.19	3.86	2.48	1.42	2.10	3.96	3.38	2.16	0.00	2.27	2.19	3.39	2.65	2.98	2.02	2.92	2.36
BCL-10	4.12	3.72	2.39	2.81	2.09	3.85	4.17	3.13	2.27	0.00	3.00	3.99	1.99	3.95	3.17	3.22	3.09
JAYCES01	2.79	3.17	2.38	1.66	2.58	3.16	2.81	1.94	2.19	3.00	0.00	2.71	2.43	2.94	1.59	2.72	1.37
JAYCES02	2.56	2.17	3.01	3.25	3.08	2.32	2.73	2.64	3.39	3.99	2.71	0.00	3.04	2.58	2.74	2.04	2.96
FAHFIG	3.54	2.98	2.07	2.84	2.04	3.05	3.57	3.11	2.65	1.99	2.43	3.04	0.00	3.40	2.82	2.13	2.14
KIHZOR	0.91	2.37	3.60	2.87	3.60	2.63	1.41	2.47	2.98	3.95	2.94	2.58	3.40	0.00	2.62	3.12	2.75
KIHZIL	2.40	2.83	2.24	1.23	2.54	2.81	2.45	1.58	2.02	3.17	1.59	2.74	2.82	2.62	0.00	2.72	1.87
1Z95	3.37	2.77	2.30	3.02	2.13	2.90	3.51	2.76	2.92	3.22	2.72	2.04	2.13	3.12	2.72	0.00	2.49
JAYCES	2.76	3.15	2.68	2.09	2.56	3.18	2.81	2.14	2.36	3.09	1.37	2.96	2.14	2.75	1.87	2.49	0.00

Table S4. Dihedral Angles and Internuclear Distances Indicating Potential Intramolecular Hydrogen Bonds in BCL Conformers Derived from CCDC, PDB, and Quantum Chemical Calculations

	**JAYCES01**	**JAYCES02**	**FAHFIG**	**KIHZOR**	**KIHZIL**	**1Z95**	**JAYCES**
C14-C13-S-C12	89.8	-103.8	104.6	-74.3	26.4	-84.8	89.5
C13-S-C12-C10	-88.3	72.5	-80.4	-44.7	-141.4	135.1	-87.4
S-C12-C10-C9	-64.2	-65.6	-67.2	83.2	70.8	-66.5	-64.9
C12-C10-C9=O	-51.3	-53.1	-48.9	50.9	31.0	-62.9	-49.9
C12-C10-C9-N	130.2	128.8	133.8	-131.9	-153.3	111.8	131.5
C10-C9-N-H	4.9	-1.9	-5.5	3.1	2.2	-1.7	-10.9
C10-C9-N-C2	-175.1	178.2	171.2	179.3	-175.1	-178.5	-175.3
C9-N-C2-C3	-28.3	-164.4	-177.1	169.1	168.3	177.9	-27.9
N-H...OH	2.15	2.10	2.11	2.04	2.19	2.02	2.13
N-H...OS	3.29	3.79	3.35	4.51	3.74	2.52	3.11
O-H...OS	2.64	3.01	3.73	2.81	3.74	3.65	4.01
O-H...OC	3.59	4.21	4.11	4.47	4.21	3.77	4.05

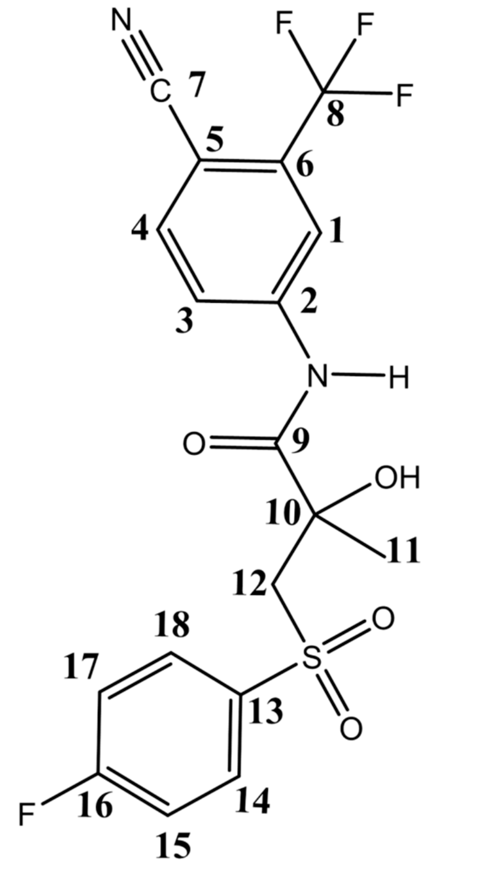

	BCL-1	BCL-2	BCL-3	BCL-4	BCL-5	BCL-6	BCL-7	BCL-8	BCL-9	BCL-10
C14-C13-S-C12	-70.4	-65.6	-89.8	-89.9	87.0	-35.5	-39.0	92.0	87.2	69.2
C13-S-C12-C10	-65.1	-64.0	178.8	179.9	-159.1	-76.1	-75.9	169.3	-159.4	84.3
S-C12-C10-C9	73.6	75.0	70.3	70.7	-175.8	56.6	55.2	-91.5	-175.9	178.9
C12-C10-C9=O	48.1	51.1	53.8	53.7	55.3	81.3	80.3	-122.5	54.9	59.9
C12-C10-C9-N	-132.9	-128.7	-128.1	-128.2	-125.6	-95.2	-96.2	58.1	-126.1	-120.3
C10-C9-N-H	1.6	1.6	1.3	1.6	2.8	2.4	1.7	-4.9	2.9	1.6
C10-C9-N-C2	171.6	166.0	176.7	176.0	-178.4	163.0	164.6	-178.4	-178.1	-178.8
C9-N-C2-C3	-172.1	16.0	1.7	-177.5	1.3	17.2	-166.5	-6.5	-179.6	0.1
N-H...OH	2.01	2.01	2.03	2.03	1.95	2.10	2.08	3.86	1.95	1.95
N-H...OS	3.75	3.69	3.52	3.48	4.64	2.45	2.46	1.76	4.64	4.63
O-H...OS	1.82	1.82	1.87	1.87	1.90	3.81	3.82	5.07	1.89	1.90
O-H...OC	3.99	4.02	4.04	4.04	4.28	3.83	3.84	1.88	4.28	4.28
